# Awareness of complications of diabetes mellitus and its associated factors among type 2 diabetic patients at Addis Zemen District Hospital, northwest Ethiopia

**DOI:** 10.1186/s13104-019-4637-x

**Published:** 2019-09-18

**Authors:** Yitayeh Belsti, Yonas Akalu, Haileab Fekadu, Yaregal Animut

**Affiliations:** 1 Department of Physiology, College of Medicine and Health Sciences, University of Gondar, Gondar, Ethiopia; 2 Department of Epidemiology and Biostatistics, Institute of Public Health, College of Medicine and Health Sciences, University of Gondar, Gondar, Ethiopia

**Keywords:** Diabetes complication, Type 2 diabetics, Ethiopia

## Abstract

**Objectives:**

Awareness about complications is important to reduce diabetes related morbidity and mortality. This study aimed to assess awareness of diabetes complications and associated factors among type 2 diabetic patients. Institution based cross sectional study was conducted from April to June, 2019, using simple random sampling technique, and interviewer administered questionnaires.

**Results:**

The mean age was 41 ± 1.46 years and 56% were females. Awareness regarding diabetic complications was 48.5% with 95% CI (43.3, 52.7%). Male sex (AOR: 4.67, 95% CI (2.53, 8.61)), age of 31–45 years (AOR: 7.30, 95% CI (3.10, 17.17)), 46–70 years old (AOR: 15.02, 95% CI (6.11, 36.92)), read and write (AOR: 3.79, 95% CI (1.78, 8.06)), primary school (AOR: 9.58, 95% CI (3.26, 28.18)), high school and above (AOR: 7.46, 95% CI (3.02, 18.44)), NGO employee (AOR: 7.24, 95% CI (2.68, 19.53)), having a family history of DM (AOR: 5.55, 95% CI (2.53, 12.20)); income of 1001–1500 (AOR: 3.22, 95% CI (1.28, 8.10)), 1501–2500 (AOR: 11.73, 95% CI (4.32, 31.81)) and > 2500 Ethiopian birr (AOR: 7.18, 95% CI (1.70, 30.28)) ≥ 2500 ETB (AOR: 7.18, 95% CI (1.70, 30.28)) were significantly associated with good awareness. To improve patient’s awareness on DM complications providing health education for type 2 diabetic patients is crucial.

## Introduction

Diabetes mellitus (DM), a group of metabolic disorders characterized by high blood glucose levels over a prolonged period, is increasing rapidly in both developed and developing countries [1]. Unless appropriate intervention is taken, it is expected that there will be at least 350 million people in the world with type 2 diabetes by the year 2030 [2]. Type 2 diabetes will be the predominant public health problem in Africa which is expected to be 28 million by 2030 [3] and 41.6 million in 2045 [4]. According to the International Diabetes Federation (IDF) report, there were 2, 652,129 cases of diabetes in Ethiopia in 2017 making it the first among the top five countries of Africa for a number of people with diabetes with the age of 18–99 years [4].

Most consequences of diabetes result from its complications which include: retinopathy, diabetic foot, renal complications, stroke, heart complications, neuropathy, hypertension, and sexual dysfunction [5]. Prevalence of diabetes and its complications are increasing alarmingly which brought a heavy burden to patients and health system [5]. A systematic review and meta-analysis in the Republic of Ireland 1998–2015 show that the prevalence of diabetic complications is increasing [6]. Studies done in Ethiopia showed that prevalence of visual disturbance, neuropathy, nephropathy and diabetic foot ulcer were 33.8%, 29.5%, 15.7% and 13.6% respectively [7, 8]. More than (59.7%) half of diabetic patients were found to have been affected by one or more of the diabetic complications [9].

Most DM complications are highly preventable through increasing awareness [10, 11]. Creating awareness about the disease, treatment, and its complications is the first step in managing disease and furthermore to facilitate prevention and control activities [11–13]. In addition, adherence to treatment requires awareness about nature and complication of the disease [12, 14–16]. However, Lack of awareness on diabetes complications contributes to high rates of complications [17].

In Ireland awareness rates of ischemic heart disease and stroke among diabetic population was 89.2 and 82.8%, respectively [18], while that of Pakistan was 50% [13]. According to the IDF report most Ghanaians had lack of awareness about diabetic complications [16]. In a study in Pakistan has shown that living in urban was associated with better knowledge [19], whereas in India, being male, family history of diabetes mellitus, higher education status and executive jobs were predictor of better awareness [12]. Health education and counseling at diabetic clinic during their follow up, and repeated media exposures provide key messages on diabetes management and prevention of its complications [12]. There is no published data regarding the awareness of diabetic complications and its predictors in Ethiopia. That is why this study was conducted to assess awareness of diabetes complication and its associated factor among type 2 diabetes patients at Addis Zemen District Hospital, northwest Ethiopia.

## Main text

### Patients and methods

#### Study design, area, and period

Institution-based cross-sectional study design was employed from April 02, 2019 to June 02, 2019 at Addis Zemen District Hospital, northwest Ethiopia.

#### Source and study population

All type 2 diabetic patients of Addis Zemen Hospital were the source population and all type 2 diabetic patients who came to diabetic clinic during the 3 months of data collection period were the study population.

#### Sample size determination and sampling technique

The sample size was calculated by using a single population proportion formula with assumptions; p = 50% (as there was no previous study in Ethiopia), 95% level of confidence and 5% margin of error. Then sample size became 384. After adding 5% oversampling, the minimum calculated sample was 404. Computer generated simple random sampling technique was used to select the study participants.

#### Inclusion and exclusion criteria

All type 2 diabetic patients who were on medication for more than 1 year and ≥ 18 years old were included. All type 2 diabetes mellitus patients who were seriously ill and health professionals were excluded.

#### Variables of the study

*Dependent variable* Awareness.

*Independent variables* Age, sex, residence, marital status, occupation, educational status, income, duration since diagnosis as diabetic, family history DM.

#### Operational definitions

*DM complications* presence of one or more of complications on DM patients such as retinopathy, diabetic foot, renal complications, stroke, heart complications, teeth decay, neuropathy, hypertension, and sexual dysfunction [20].

*Rural residence* settling in country side outside of big cities or towns in Ethiopia are referred as rural residents [21].

*Family history of DM* having at least one‐first‐degree relative with diabetes [22].

#### Data collection instrument and procedure

Pretested structured interviewer-administered questionnaire, which is adapted from different literatures, was used to collect the data [15, 23–26]. The questioner contains 28 diabetes complications related awareness items. The possible correct answers for assessing awareness of diabetes complications were 28. The awareness of the patient was calculated by summating the correct answers and calculating the mean value as 15 with minimum and maximum correct answers of 5 and 25 respectively. The participant who mentioned less than mean [15] correct answers grouped as have no awareness. The participant who mentioned ≥ 15 correct answers grouped as having awareness. The questionnaire was prepared in English first and translated to local language and then, re translated back to English by another person to check its consistency and wording.

#### Data quality management/control

Training was given for data collectors and supervisors about the aim of the study, data collection procedure and ethical issues. Validity was checked by doing pretest on 60 type 2 DM patients at University of Gondar Hospital (out of the study area). Modification of the tool was made based on the pre-test result. The Cronbach’s alpha scale for awareness questions was done for all questions and it was greater than 0.7, which is acceptable. Close supervision was made during data collection. Data clean up and crosschecking was also done before analysis.

#### Data analysis procedure

The data were checked for completeness and entered to Epi Info version 7 and were exported to SPSS Version 20 for analysis. Descriptive statistics such as frequencies and percentage were used. A binary logistic regression was used to identify predictors of awareness on DM complications. On bivariable analysis, variables with a p-value < 0.2 were entered to multivariable logistic regression model. p ≤ 0.05 were used to declare statistically significant variables in the final model.

### Results

#### Socio-demographic characteristics of respondents

The study was conducted on 402 study participants. Fifty-six percent patients were female. The mean age was 41 (SD ± 1.46) years. Concerning age, 150 (37.3%) of the respondents were aged 46 to 70 years. Majority 91 (22.6%) had an educational level of high school and above. Over one-third (33.8%) of the respondents had a monthly income of below 500 Ethiopian birr. One hundred and eighteen (29.4%) study participants had a family history of diabetic mellitus (Table 1).

**Table 1:** Socio demographic characteristics of type 2 adult DM patients at Addis Zemen District Hospital, Gondar, Ethiopia, (N = 402)

Variables	Frequency	Percent (%)
Sex		
Male	177	44.0
Female	225	56.0
Age		
18–30	134	33.3
31–45	118	29.4
46–70	150	37.3
Level of education		
Cannot write and read	110	27.4
Read and write	124	30.8
Primary	77	19.2
High school and above	91	22.6
Marital status		
Married	202	50.2
Divorced	37	9.2
Widowed	49	12.2
Single	114	28.4
Occupation		
Farmer	85	21.1
Government worker	46	11.4
Merchant	104	25.9
Housewife	77	19.2
NGO worker	90	22.4
Religion		
Orthodox	304	75.6
Muslim	68	16.9
Protestant	30	7.5
Ethnicity		
Amhara	348	86.6
Kimant	35	8.7
Tigrie	19	4.7
Residence		
Rural	159	39.6
Urban	243	60.4
Duration of DM		
(1–5)	270	67.2
(6–10)	93	23.1
> 10	39	9.7
Type of medication they use		
Oral	133	33.1
Injectable	218	54.2
Both	51	12.7
Family history of DM		
Yes	118	29.4
No	284	70.6
Income		
< 500	156	38.8
500–1500	68	16.9
1501–2500	86	21.4
> 2500	92	22.9
Have you been informed about complications of diabetes?		
Yes	301	74.9
No	101	25.1
Where did you get information about DM complications?		
Health worker	253	62.9
Friend/parent	27	6.7
TV/radio	19	4.7
Other^a^	2	0.5

*DM* diabetes mellitus, *N* number, *TV* television

^a^Reading books

#### Awareness about complications of DM

In this study, 195 (48.5%) of the diabetic patients had awareness of DM complications with 95% CI (43.3, 52.7). Over two-third (62.9%) of patients were informed about diabetes complications from health professionals. Most of the study participants 372 (92.5%) had knowledge of dietary modification to prevent diabetes complications. Three hundred sixty-seven (91.3%) knew about complication related risk factor of alcohol and cigarette smoking. Diabetic foot 297 (73.9%) was the most known complication of DM followed by eye complications 292 (72.6%), and heart complications 254 (63.2%) (Table 2).

**Table 2:** Awareness on DM complications among type 2 DM patients at Addis Zemen Hospital, Ethiopia

Variables	Categories	Number	Percent
What is the normal fasting blood sugar level?	< 70 mg/dl	38	9.5
	70–110 mg/dl	77	19.2
	> 126 mg/dl	6	1.5
	Don’t know	281	69.9
What are the most common symptoms of high blood sugar?	Increased thirst	309	76.9
	Frequent urination	295	73.4
	Blurring of vision	130	32.3
	Weakness	185	46
	Dry mouth	102	25.4
	Confusion	30	7.5
What are the most common symptoms of low blood sugar?	Palpitation	157	39.1
	Tremor	220	54.7
	Sweating	238	59.2
	Blurring of vision	107	26.6
	Decreased coordination	62	15.4
Which of the following complications can happen when diabetes is not well controlled?	Diabetic foot	297	73.9
	Eye complications	292	72.6
	Heart complications	254	63.2
	Neuropathy	217	54.0
	Renal complications	216	53.7
	Stroke	157	39.1
	Teeth decay	130	32.3
	Hypertension	142	35.3
	Sexual dysfunction	102	25.4
Can dietary modification prevent diabetic complication?	Yes	372	92.5
	No	30	7.5
Can Stop smoking/and alcohol stopping prevent diabetic complication?	Yes	367	91.3
	No	35	8.7
Is physical work or exercise help to prevent diabetes complication?	Yes	343	85.3
	No	59	14.7
If you are beginning to have a low blood glucose reaction, you should?	1. Exercise	16	4.0
	2. Lie down and rest	85	21.1
	3. Drink some juice	193	48.0
	4. Take rapid-acting insulin	108	26.9
What you should do when your blood sugar is raised?	1. Dietary modification	87	21.6
	2. Physical exercise	10	2.5
	3. Lowering stress	79	19.7
	4. Take insulin	226	56.2

*DM* diabetes mellitus

#### Factors associated with awareness of diabetes mellitus complications among DM patients

All independent variables were tested for crude association with awareness by binary logistic regression. However, after adjusting for potential confounders in the multivariable analysis, being; male (AOR: 4.67, 95% CI (2.53, 8.61)), in the age of 31–45 years (AOR: 7.30, 95% CI (3.10, 17.17)), 46–70 years old (AOR: 15.02, 95% CI (6.11, 36.92)), educational level of read and write (AOR: 3.79, 95% CI (1.78, 8.06)), primary school [1–8] (AOR: 9.58, 95% CI (3.26, 28.18)), high school and above (AOR: 7.46, 95% CI (3.02,18.44)), being NGO employee (AOR: 7.24, 95% CI (2.68,19.53)), having a family history of DM (AOR: 5.55, 95% CI (2.53,12.20)); income of 1001–1500 (AOR: 3.22, 95% CI (1.28,8.10)), 1501–2500 (AOR: 11.73, 95% CI (4.32, 31.81)) and > 2500 Ethiopian birr (AOR: 7.18, 95% CI (1.70,30.28)) were significantly (p < 0.05) associated with a good knowledge (Table 3).

**Table 3:** Factors associated with awareness of DM complications among DM patients at Addis Zemen Hospital, Ethiopia

Variable	Knowledge status (n = 402)		OR (95% CI)	
	Poor N (%)	Good N (%)	COR	AOR
Sex				
Male	64 (36.2)	113 (63.8)	3.08 (2.04, 4.64)	4.67 (2.53, 8.61)***
Female	143 (63.6)	82 (36.4)	1	1
Age				
18–30	100 (74.6)	34 (25.4)	1	1
31–45	59 (50.0)	59 (50.0)	2.94 (1.73, 5.00)	7.30 (3.10, 17.17)***
46–70	48 (32.0)	102 (68.0)	6.25 (3.72, 10.49)	15.02 (6.11, 36.92)***
Level of education				
Cannot write and	80 (74.1)	28 (45.5)	1	1
Read and write	75 (49.0)	78 (51.0)	2.97 (1.74, 5.07)	3.793 (1.78, 8.06)**
1–8	20 (40.0)	30 (60.0)	4.29 (2.11, 8.73)	9.59 (3.26, 28.18)***
High school	32 (35.2)	59 (59)	5.27 (2.86, 9.68)	7.46 (3.02, 18.44)***
Occupation				
Farmer	55 (64.7)	30 (35.3)	1	1
Government worker	15 (32.6)	31 (67.4)	3.79 (1.77, 8.10)	0.96 (0.31, 2.95)
Merchant	47 (45.2)	57 (54.8)	2.22 (1.23, 4.01)	0.72 (0.29, 1.81)
House wife	43 (55.8)	34 (44.2)	1.45 (0.78, 2.73)	2.93 (1.13, 7.56)
NGO worker	47 (52.2)	43 (47.8)	1.68 (0.91, 3.08)	7.24 (2.68, 19.53)***
Residence				
Rural	99 (62.3)	60 (37.7)	1	1
Urban	108 (44.4)	135 (55.6)	2.06 (1.37, 3.10)	1.76 (0.13, 3.11)
Duration of DM				
< 5	152 (56.3)	118 (43.7)	1	1
5–10	41 (44.1)	52 (55.9)	1.63 (1.05, 2.63)	0.89 (0.36, 2.22)
> 10	14 (35.9)	25 (64.1)	2.30 (1.15, 4.62)	1.25 (0.47, 3.29)
Family history of DM				
Yes	19 (16.1)	99 (83.9)	10.20 (5.89, 17.67)	5.55 (2.53, 12.20)***
No	188 (66.2)	96 (33.8)	1	1
Income				
< 500	93 (68.4)	43 (31.6)	1	1
501–1000	44 (64.7)	24 (35.3)	1.18 (0.64, 2.18)	1.85 (0.75, 4.57)
1001–1500	42 (48.8)	44 (51.2)	2.27 (1.30, 3.95)	3.22 (1.28, 8.10)*
1501–2500	21 (25.6)	61 (74.4)	6.28 (3.40, 11.60)	11.73 (4.32, 31.81)***
> 2500	7 (23.3)	23 (76.7)	7.11 (2.83, 17.83)	7.18 (1.72, 30.28)**

*COR* crude odds ratio, *AOR* adjusted odds ratio, *CI* confidence interval, *DM* diabetes mellitus, *N* number

* p-value < 0.05, ** p-value < 0.01, *** p-value < 0.001, 1 = references

### Discussions

This study has provided data about the awareness of complications of diabetes mellitus among diabetic patients at Addis Zemen Hospital, northwest Ethiopia. Comprehensive assessment of the awareness of 402 patients on DM complications showed that less than half of participants had awareness. This finding is consistent with the study conducted in Ghana (40%) [25], Bangladesh (42%) [26], and in Pakistan [24]. On the contrary, it is not in line with a study conducted in Saudi Arabia on awareness of diabetic complications which reported that 80% of participants were aware about complications of diabetes. The reason for the difference may be because of a difference in socioeconomic conditions, cultural beliefs and habits, as studies showed that differences in such variables had an impact on the pattern of awareness on diabetic complications [27]. In the current study, age, sex, educational level, occupation, and family history of DM were significantly associated with awareness of DM complications. This finding, except for age, is congruent with the study done in India [12]. In the current study those who were male were 4.6 times more likely to have awareness than females. Consistently, a study in Pakistan showed that being male was predictor of better awareness [24]. The justification for this finding might be due to cultural influence which allows females to spend their time in house, but males spent most of their time outside the home that gave them more chance to acquire more information and to attend different meetings and conferences. Age was also significantly associated with awareness of DM complications which is similar with the study done in Bangladesh [26]. Those 31–45 years old were 7.3 more likely to have awareness than those with age 15–30 years old. As age increases, they get more counseling and health education during their follow-up at the diabetic clinic and from formal and non-formal education. A higher level of education was a significant predictor of better awareness, this finding coincides with other studies [12, 24]. Those with educational level of read and write, 1-8 and, high school and above were 3.7, 9.5 and 7.4 times more aware of DM complications, respectively than those who cannot read and write. As individuals learn more, the chance of gaining information about DM complications from different sources will increase. In addition, those individuals with a high level of education can read different medical books. Another interesting finding of this study was the association between occupation and patient knowledge of diabetic complications. NGO employees were 7 times more likely to have awareness about diabetic complications than those who were farmers. These results agree with findings of Obirikorang et al. [24]. This is because NGO employees are mostly linked to different institution especially health institutions which create them a favorable condition to gain information about complication of DM. This study also found a significant association between income and awareness on diabetic Complications. Those patients with an income of greater than 2500 were aware of DM complications than those with income of less than < 500. This finding is supported by other study [24]. It is clear that as income increases, individuals can access or buy any electronic media like TV, which is one means of gaining information. Another important finding of this study was patients with a family history of DM were five times more likely to have awareness on DM complications than the counterparts. This finding is consistent with the study done India [12]. This is because, they learn from their family experience. Most of diabetic patients have no awareness on diabetic complications. Age, sex, educational level, occupation, and family history of DM were significant predictors of awareness of DM complications. Health education on DM complications for type 2 diabetes patients by health professionals and through mass media should be promoted.

## Limitations of the study

During assessment of knowledge of patients on DM chronic complications, even though the interviewers carried out it carefully, respondents may have replied socially acceptable responses which may cause an overestimation of awareness of study participants.

## Data Availability

The data will be available upon request from the corresponding author upon request.

## Abbreviations

DM: diabetic mellitus
NGO: non-governmental organization
AOR: adjusted odds ratio
CI: confidence interval
COR: crude odds ratio
SPSS: statistical package for social sciences
